# Flagellin-independent effects of a Toll-like receptor 5 polymorphism in the inflammatory response to *Burkholderia pseudomallei*

**DOI:** 10.1371/journal.pntd.0007354

**Published:** 2019-05-08

**Authors:** Amy K. Dickey, Narisara Chantratita, Sarunporn Tandhavanant, Deirdre Ducken, Lara Lovelace-Macon, Sudeshna Seal, Johanna Robertson, Nicolle D. Myers, Sandra Schwarz, Mark M. Wurfel, Susanna Kosamo, T. Eoin West

**Affiliations:** 1 Department of Medicine, University of Washington, Seattle, Washington, United States of America; 2 Department of Microbiology and Immunology, Faculty of Tropical Medicine, Mahidol University, Bangkok, Thailand; 3 Mahidol-Oxford Tropical Medicine Research Unit, Faculty of Tropical Medicine, Mahidol University, Bangkok, Thailand; 4 Interfaculty Institute of Microbiology and Infection Medicine, University of Tübingen, Tübingen, Germany; 5 International Respiratory and Severe Illness Center, University of Washington, Seattle, Washington, United States of America; University of Texas Medical Branch, UNITED STATES

## Abstract

**Background:**

Toll-like receptors (TLRs) are sentinel receptors of the innate immune system. TLR4 detects bacterial lipopolysaccharide (LPS) and TLR5 detects bacterial flagellin. A common human nonsense polymorphism, *TLR5*:c.1174C>T, results in a non-functional TLR5 protein. Individuals carrying this variant have decreased mortality from melioidosis, infection caused by the flagellated Gram-negative bacterium *Burkholderia pseudomallei*. Although impaired flagellin-dependent signaling in carriers of *TLR5*:c.1174C>T is well established, this study tested the hypothesis that a functional effect of *TLR5*:c.1174C>T is flagellin-independent and involves LPS-TLR4 pathways.

**Methodology/Principal findings:**

Whole blood from two independent cohorts of individuals genotyped at *TLR5*:c.1174C>T was stimulated with wild type or aflagellated *B*. *pseudomallei* or purified bacterial motifs followed by plasma cytokine measurements. Blood from individuals carrying the *TLR5*:c.1174C>T variant produced less IL-6 and IL-10 in response to an aflagellated *B*. *pseudomallei* mutant and less IL-8 in response to purified *B*. *pseudomallei* LPS than blood from individuals without the variant. *TLR5* expression in THP1 cells was silenced using siRNA; these cells were stimulated with LPS before cytokine levels in cell supernatants were quantified by ELISA. In these cells following LPS stimulation, silencing of *TLR5* with siRNA reduced both TNF-α and IL-8 levels. These effects were not explained by differences in *TLR4* mRNA expression or NF-κB or IRF activation.

**Conclusions/Significance:**

The effects of the common nonsense *TLR5*:c.1174C>T polymorphism on the host inflammatory response to *B*. *pseudomallei* may not be restricted to flagellin-driven pathways. Moreover, TLR5 may modulate TLR4-dependent cytokine production. While these results may have broader implications for the role of TLR5 in the innate immune response in melioidosis and other conditions, further studies of the mechanisms underlying these observations are required.

## Author summary

Toll-like receptors (TLRs) are important receptors of the innate immune system. TLR4 detects bacterial lipopolysaccharide (LPS) and TLR5 detects bacterial flagellin. A common human polymorphism in *TLR5* encodes a shortened protein and blunts the immune response to flagellin. Individuals carrying this variant have decreased mortality from melioidosis, infection caused by the flagellated Gram-negative bacterium *Burkholderia pseudomallei*. The mechanism of protection is not known. We tested the hypothesis that the observed effect of the polymorphism is independent of flagellin and involves LPS-TLR4 pathways. We found that blood from individuals carrying the polymorphism produced lower levels of cytokines IL-6 and IL-10 in response to an aflagellated *B*. *pseudomallei* mutant and less IL-8 in response to purified *B*. *pseudomallei* LPS than blood from individuals without the variant. We further observed that in THP1 cells stimulated with LPS, silencing of *TLR5* with siRNA reduced levels of both TNF-α and IL-8. These effects were not explained by differences in *TLR4* mRNA expression. We conclude that the effects of the *TLR5* polymorphism on the host inflammatory response to *B*. *pseudomallei* may not be restricted to flagellin-driven pathways. These results provide insights into the role of TLR5 in the innate immune response in melioidosis and other conditions.

## Introduction

Melioidosis is a tropical infection caused by the flagellated Gram-negative bacterium *Burkholderia pseudomallei* [1]. The disease commonly presents as pneumonia and sepsis in Southeast Asia and northern Australia. The mortality rate from infection with this bacterium can exceed 40% [2]. Hawn *et al*. first described a nonsense human Toll-like receptor (TLR)-5 polymorphism, *TLR5*:c.1174C>T (rs5744168), that eliminates both the transmembrane and cytoplasmic signaling regions of the protein, rendering it unable to signal in response to flagellin [3]. Approximately 10% of white individuals of European ancestry [3] and Thai individuals [4] carry the minor T allele. While we have previously found that while the *TLR5*:c.1174C>T polymorphism does not affect susceptibility to infection with melioidosis [4], the variant is associated with significantly improved survival of individuals with culture-proven melioidosis [5, 6]. Furthermore, we have studied the effect of the variant on cytokine expression induced by stimulation of whole blood *ex vivo* with *B*. *pseudomallei*. We have found that IL-6 and IL-10 levels were reduced in white North American carriers of the variant allele while IL-10 levels were reduced in Thai carriers [5]. Initially, these associations would seem to involve a mechanism including flagellin, as this is the bacterial motif that activates TLR5. However, we have previously reported that lipopolysaccharide (LPS), and not flagellin, is primarily responsible for the whole blood innate immune response to killed *B*. *pseudomallei*, highlighting the critical role of TLR4 in the host response of this organism [7]. Moreover, multiple TLR family members form heterodimers to facilitate innate immune signaling, and TLR5 in particular has been reported to bind TLR4 in transfected cells stimulated with flagellin [8]. We therefore considered the possibility that a TLR5/TLR4 signaling interaction may underlie the effect of the *TLR5*:c.1174C>T polymorphism on inflammatory responses and survival in *B*. *pseudomallei* infection. In this study, we tested the hypothesis that TLR5 can modulate the flagellin-independent, TLR4-dependent innate immune response.

## Materials and methods

### Bacteria

To construct a markerless in-frame deletion of *B*. *pseudomallei* flagellin, *fliC* (BP1026b_I3555), primers were designed to amplify approximately 800 bp regions up- and downstream of the gene including the first and last four codons of *fliC*, respectively. The up- and downstream fragments were joined by an overlap sequence using splicing by overlap extension as described previously [9]. The deletion construct was cloned into the suicide vector pMo130, which carries a kanamycin resistance gene for the selection of transformants and a *sacB* gene as a counter-selectable marker [10]. Next, the plasmid was transferred from *E*. *coli* SM17 λpir to *B*. *pseudomallei* 1026b by mating. Transconjugants were selected on LB plates containing irgasan (25 μg/ml) and kanamycin (300 μg/ml) and streaked onto LB agar supplemented with 15% sucrose to select for transformants that resolved the merodiploid state. Colonies were screened for excision of the plasmid by replica plating on LB/kanamycin (500 μg/ml) and LB plates. The deletion of *fliC* in kanamycin sensitive colonies was verified by PCR and swimming motility assays. *B*. *pseudomallei* 1026b and *B*. *pseudomallei* Δ*fliC* were grown, shaking, in LB broth for six hours in air at 37°C and heat-killed before use in *in vitro* assays [5].

### HEK293 cell transfection and stimulation

To test *B*. *pseudomallei* Δ*fliC*, HEK293 cells were used as previously reported with some modifications [11]. Cells were cultured in a 96 well flat-bottomed tissue culture plate at 15–50 × 10^3^ cells/well in DMEM plus 10% fetal bovine serum (FBS) and 1% L-glutamine at 37°C under 5% CO_2_. The following day cells at 60–80% confluency were transiently transfected using PolyFect transfection reagent (Qiagen, Valencia, CA). NF-κB-ELAM firefly luciferase 80 ng/well was transfected with β-actin-*Renilla* luciferase 8 ng/well, and hu*TLR5* (cloned into pEF6, a gift from Dr. Thomas R. Hawn [3]) 175ng/well. Cells were immediately stimulated with IL-1β, flagellin from *S*. Typhimurium strain SL1344 deficient in *flgM* (engineered to overproduce flagella, a gift from Kelly Smith, University of Washington [12]), *B*. *pseudomallei* 1026b or *B*. *pseudomallei* Δ*fliC* and incubated for 24 hours. Purity of the flagellin was confirmed by limulus amebocyte lysate assay and SDS-PAGE. Cells were then lysed with passive lysis buffer (Promega, Madison, WI) and NF-κB activation was determined in 10 μl of lysate by the ratio of firefly to *Renilla* luciferase light emission using the Dual Luciferase Reporter System (Promega).

For TLR4/TLR5 co-transfections, HEK293 cells stably transfected with MD-2, CD14, and TLR4 were obtained from Invivogen (Catalog # 293-htlr4md2cd14). Cells were cultured in a 96-well flat-bottomed tissue culture plate at 50 × 10^3^ cells/well in cell growth media (DMEM, 4.5 g/l glucose, 50 U/ml penicillin, 50 μg/ml streptomycin, 100 μg/ml Normocin, 10 μg/ml blasticidin, 50 μg/ml Hygromycin B Gold, plus 10% fetal bovine serum (FBS) and 1% L-glutamine). The following day cells were transiently transfected using PolyFect transfection reagent (Qiagen, Valencia, CA) and the following DNA: NF-κB-ELAM firefly luciferase 50 ng/well, HSV thymidine kinase-*Renilla* luciferase (pRL-TK, Promega) 5 ng/well, and hu*TLR5* (cloned into pEF6) and empty pEF6 vector at 1–10 ng/well as indicated. The transfection complexes were allowed to incubate with the cells for 24 hours. The following morning, the media in the wells was aspirated and the cells were then stimulated with ultrapure *Escherichia coli* O111:B4 LPS (List Biologicals, Campbell, CA) or ultrapure *S*. Typhimurium flagellin (Invivogen) suspended in cell growth media. After four hours, cells were lysed with passive lysis buffer and NF-κB activation was determined as above.

### Whole blood immunoassays

For *ex vivo* whole blood immunoassay studies, 380 μl of fresh whole blood in citrate mixed 1:1 with RPMI media was added to pre-prepared plates containing 20 μl of stimulants. Studies were performed at Harborview Medical Center (HMC) in Seattle, USA, and in Sunpasitthiprasong Hospital, Ubon Ratchathani, Thailand as previously described [5, 13]. For the HMC study the stimulant analyzed was log phase heat-killed *B*. *pseudomallei* 1026b Δ*fliC* 2.5 x 10^6^ CFU/ml. For the Sunpasitthiprasong Hospital study the stimulants analyzed were *B*. *pseudomallei* K96243 LPS 10 ng/ml, *B*. *pseudomallei* V688 LPS 10 ng/ml, *B*. *pseudomallei* 558 LPS 10 ng/ml, *S*. Typhimurium strain SL1344 deficient in *flgM* flagellin 500 ng/ml, Pam_3_CSK_4_ 100 ng/ml (Invivogen, San Diego, CA), and *E*. *coli* 0111:B4 LPS 10 ng/ml (List Biological Laboratories, Campbell, CA). *B*. *pseudomallei* LPS preparations (a gift from Bob Ernst, University of Maryland) were generated using a hot phenol/water extraction method after growth of bacteria in lysogeny broth (LB) supplemented with 1 mM MgCl_2_ at 37°C [14]. Subsequently, LPS was treated with RNase A, DNase I and proteinase K to ensure purity from contaminating nucleic acids and proteins [15]. The LPS sample was additionally extracted to remove contaminating phospholipids [16] and TLR2 contaminating proteins [17]. We have previously demonstrated the lack of TLR2 activation by *B*. *pseudomallei* LPS prepared in this fashion [11].

Plates were incubated at 37°C on a shaking incubator under 5% CO_2_ for six hours before being spun down and plasma removed and frozen. For the HMC study, plasma cytokines were subsequently assayed in duplicate using an electrochemiluminescence imager (Mesoscale Discovery). For the Sunpasitthiprasong Hospital study, cytokines were assayed in duplicate on a multiplex bead system (Luminex, Austin, TX) using reagents from R&D Systems. For each subject, a complete blood count with differential was performed in the clinical laboratory at the time of phlebotomy.

### Monocyte isolation and stimulation

Eight mL of whole blood was drawn from healthy subjects into BD vacutainer cell preparation tubes with sodium citrate (BD, Franklin Lakes, NJ) and centrifuged at 1500 RCF for 20 minutes. The PBMC layer was collected and washed twice with PBS, according to the manufacturer’s instructions. Monocytes were isolated from this preparation of PBMCs using the Miltenyi Biotec monocyte isolation kit II (Auburn, CA). With this kit, non-monocytes were indirectly magnetically labeled using a cocktail of biotin-conjugated antibodies and anti-biotin microbeads. Enriched monocytes were collected after running the preparation on LS columns, retaining all of the non-monocytes in the columns. This procedure was completed according to the manufacturer’s instructions. Isolated monocytes were plated in RPMI media plus 10% FBS in a 96 well plate, with 50,000 monocytes per well. Monocytes were stimulated with ultrapure *E*. *coli* O111:B4 LPS (List Biological Laboratories) for four hours.

### RNA extraction and quantitative RT-PCR

RNA was isolated from monocytes using the Applied Biosystems Nucleic Acid Lysis Solution and the ABI Prism 6100 Nucleic Acid PrepStation (Applied Biosystems, Grand Island, NY), according to the manufacturer’s instructions. RNA expression was determined using the SensiFAST Probe Lo-Rox One Step Kit (Bioline, Taunton, MA). For each PCR reaction, 2ul RNA, 0.5 μl Taqman primers (Life Technologies, Grand Island, NY), 5 μl SensiFAST mix, 0.1 μl reverse transcriptase, 0.2 μl RNAase inhibitor, and 2.2 μl water was added to each well of a 384 well plate. The reactions were completed using a Viia7 Real-Time PCR System (Applied Biosystems), according to the thermocycling conditions suggested by Bioline. The following commercially available Taqman primers from Applied Biosystems were used for the PCR reactions: GAPDH HS02758991_g1 and TLR4 HS00152939_m1.

RNA was isolated from transfected THP1-Dual Cells using the Promega ReliaPrep RNA Cell Miniprep System (Promega, WI), according to the manufacturer’s instructions. RNA expression was determined using the ABI High Capacity cDNA Reverse Transcriptase Kit (Applied Biosystems) and Bio-Rad SsoAdvanced Universal SYBR Green Supermix (Bio-Rad, CA). The reactions were completed using a ViiA7 Real-Time PCR System (Applied Biosystems), according to the thermocycling conditions suggested by Bio-Rad. The following commercially available primer assays from Bio-Rad were used for the PCR reactions: TLR5 (qHsaCID0009328), TLR4 (qHsaCED0037607), and UBC (qHsaCED0023867).

### DNA extraction and genotyping

For whole blood stimulation studies, DNA extraction and genotyping was performed as previously described [5]. For primary human monocyte studies, DNA was extracted from saliva using Oragene kits (DNA Genotek) and genotyping was performed using ABI TaqMan assays on an ABI Prism 7900.

### Human subjects

Fasting blood samples for whole blood stimulation were obtained from healthy white participants in a Harborview Medical Center (HMC) inflammatory response research study [5]. Similarly, healthy Thai subjects donating blood at the blood donation center at Sunpasitthiprasong Hospital in 2010 were also recruited for investigation of inflammatory responses. Enrollment criteria and blood processing for both studies has been previously described [5, 7, 13]. Blood for monocyte isolation was obtained from healthy subjects of Southeast Asian ancestry at Harborview Medical Center from whom saliva was obtained and genotyped. Subjects were between the ages of 18 and 65 years, weighed between 100 and 350 pounds, did not smoke, did not have any chronic medical conditions, were not pregnant and had not given birth within the preceding nine months, did not have symptoms of infection within the past two weeks, had not received a vaccination in the past six weeks, and did not take any anti-inflammatory, antimicrobial, or prescription (other than oral contraceptive) medication. Subjects were asked to refrain from any heavy exercise or alcohol use for 24 hours and to fast overnight before the blood draw.

### Ethics statement

The University of Washington Human Subjects Division Institutional Review Board; the Ethical Review Committee for Research in Human Subjects, Sunpasitthiprasong Hospital, Ubon Ratchathani, Thailand; and the Ethics Committee of the Faculty of Tropical Medicine, Mahidol University, Bangkok, Thailand approved the studies. All participating subjects were adults who gave written informed consent.

### Generation of recombinant *B*. *pseudomallei* flagellin

The chromosomal DNA of Bp82, a *ΔpurM* mutant of *B*. *pseudomallei* [18], was isolated using the Promega Wizard Genomic DNA Purification Kit (WI, USA). Primers 5’-TTTTGGATCCATGCTCGGAATCAACAGCAACATTAAC-3’ (forward primer) and 5’-TTTTGCGGCCGCTTATTGCAGGAGCTTCAGCACTTGC-3’ (reverse primer) [19] were designed to amplify the full-length flagellin gene. The resultant PCR amplified flagellin gene was cloned into the plasmid pCR 2.1-TOPO (CA, USA) according to the manufacturer’s protocol. This PCR construct was used to generate a N-terminal 6X-His-tag-flagellin protein under contract by NOVOprotein (Summit, NJ, USA). The endotoxin content of the recombinant flagellin was verified to be <100 EU/mg.

### TLR5 knockdown in THP1-Dual cells

THP1-Dual Cells (NF-κB-SEAP and IRF3-Lucia luciferase Reporter Monocytes, Invivogen, CA) were differentiated over a period of 72 hours with Vitamin D3 (final concentration 10 pM/ml) and plated at 50,000 cells per well in media containing D3 (10 pM/ml). The next day, cells were transfected with Silencer Select siRNA (Ambio, MA) against TLR5 (siRNA ID—s14197) or a Silencer Select Negative Control No. 1 siRNA (# 4390843) at a final concentration of 5 nM using Lipofectamine RNAiMAX transfection Reagent (Life Technologies, CA). After two days, some cells were lysed for RNA extraction and remaining cells were stimulated with either *E*. *coli* LPS at varying doses of recombinant *B*. *pseudomallei* flagellin 100 ng/ml as a positive control. The following day, supernatants were collected and assayed for SEAP using QUANTI-Blue detection reagent and IRF3 using QUANTI-Luc luciferase detection reagent according to the manufacturer’s instructions. Additional supernatants were also stored at -80°C until ready to assay. TNF-α and IL-8 concentrations were determined using DuoSet ELISA (R&D, MN) using the manufacturer’s protocol.

### Statistics

Continuous *in vitro* data expected to follow a normal distribution are reported as mean ± standard deviations; comparisons between two groups were made using the t test. Non-normal data are reported as individual values and median; comparisons between two groups are made using the rank sum test. Cytokine values were normalized to monocyte counts and log_10_ transformed before analysis by linear regression, adjusting in the Sunpasitthiprasong cohort for age, sex, and batch. For gene expression, quantitative real-time RT-PCR C_t_ values observed for each sample were normalized to the reference gene values and reported as 2^-ΔCt^. Statistics were performed with Stata 14.2 (College Station, TX). A two-sided P value ≤0.05 was considered significant.

## Results

### Carriers of *TLR5*:c.1174C>T generate reduced cytokine responses to aflagellated *B*. *pseudomallei*

**Fig 1:**
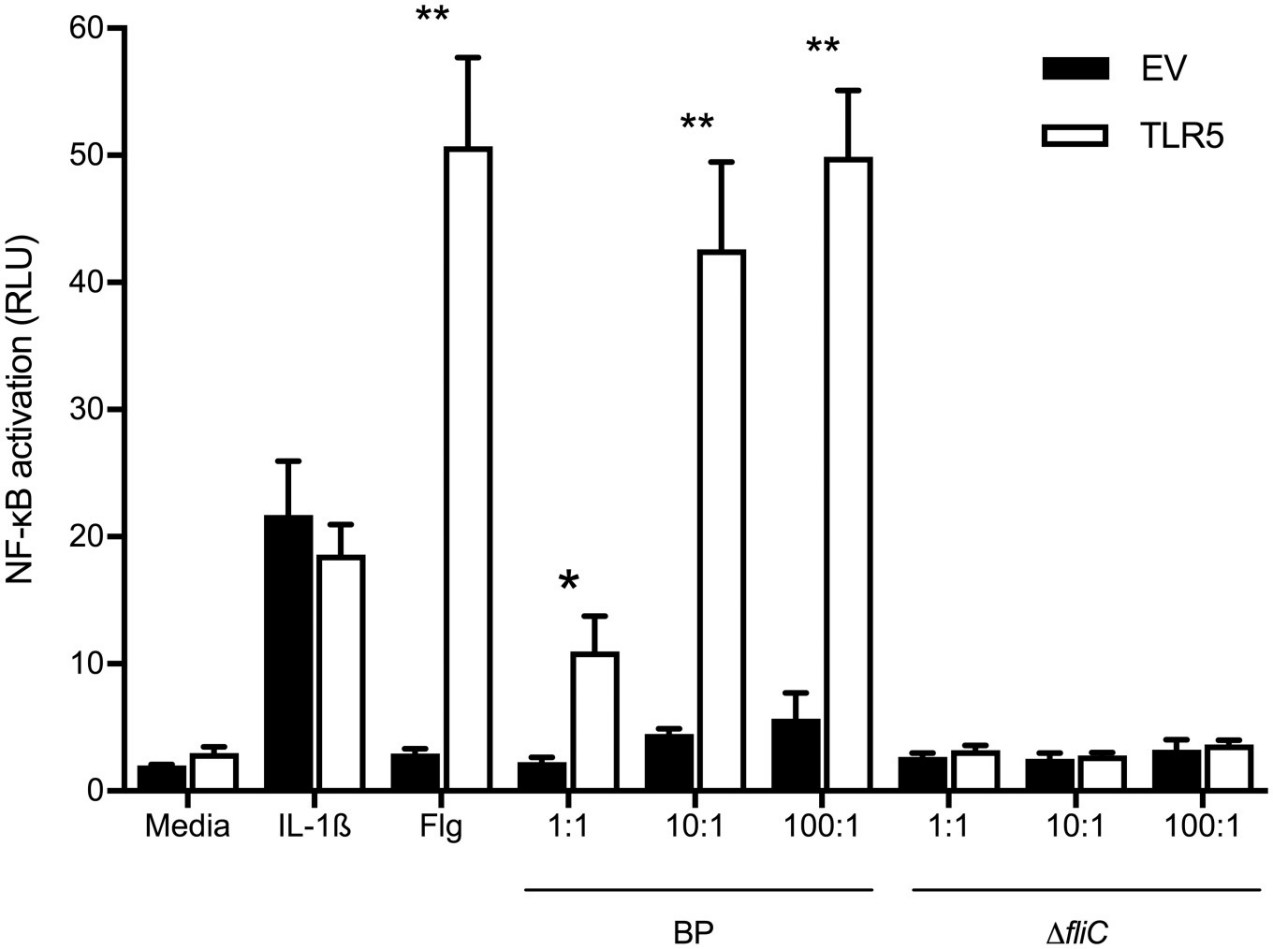
Lack of TLR5-dependent NF-κB activation by flagellin-deficient *B*. *pseudomallei*. HEK293 cells were transiently transfected with empty vector pEF6 (EV) or TLR5, NF-κB-dependent firefly ELAM luciferase, and control *Renilla* luciferase. Cells were unstimulated or stimulated with IL-1β 20 ng/ml, *Salmonella* Typhimurium flagellin 500 ng/ml (Flg), wildtype *B*. *pseudomallei* 1026b (BP) or *B*. *pseudomallei* 1026b lacking *fliC*, the gene that encodes flagellin *(*Δ*fliC*) at a range of bacteria:cell ratios for 24 hours. NF-κB activation was measured by luciferase assay (ratio of ELAM/*Renilla* luciferase signal expressed as relative light units). Mean values ± standard deviations of triplicate conditions are displayed. *, P≤0.05; **, P≤0.01 comparing TLR5-transfected to empty vector-transfected cells using the t test. One representative example of two independent experiments is shown.

**Fig 2:**
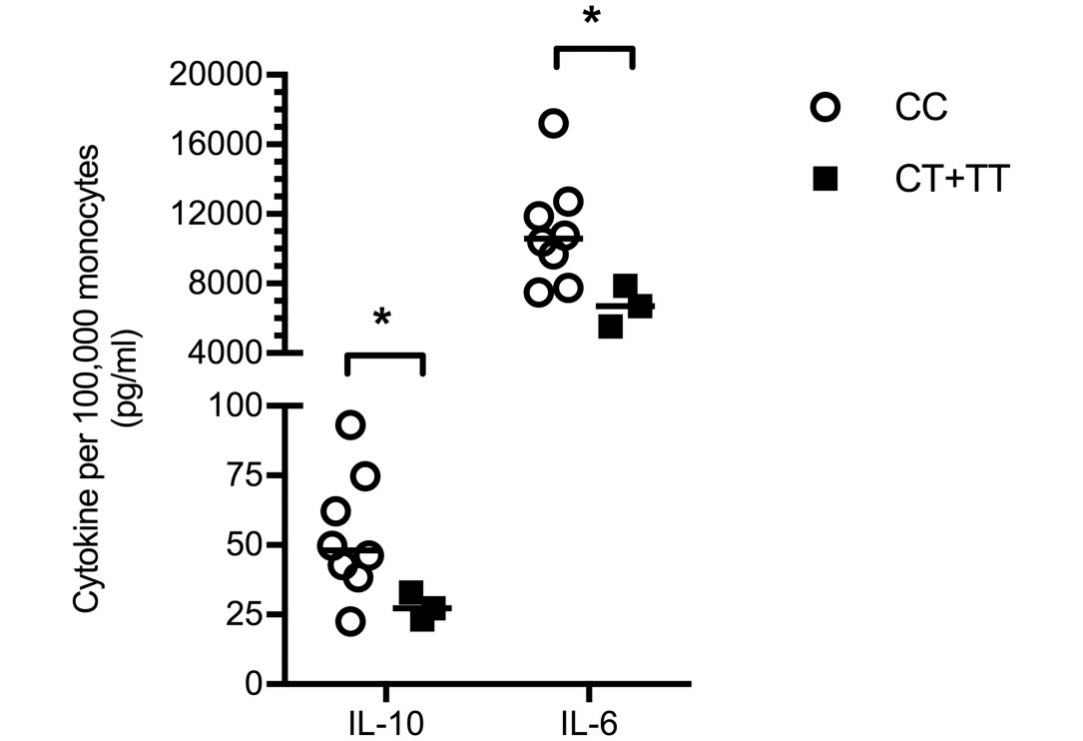
Carriers of *TLR5*:c.1174C>T generate reduced IL-10 and IL-6 responses to flagellin-deficient *B*. *pseudomallei*. Whole blood from healthy white North American subjects was stimulated with heat-killed *B*. *pseudomallei* Δ*fliC* at 2.5 × 10^6^ CFU/ml for 6 hours. IL-10 and IL-6 were measured in plasma by electrochemiluminescence assay and normalized to monocyte count. Superimposed lines represent the median values. Statistical analysis was performed by linear regression on log_10_ transformed data. *, P≤0.05. *TLR5*:c.1174C>T genotype (CC, CT, or TT) was determined for each individual by genotyping whole blood DNA. n = 8 (CC), n = 3 (CT+TT).

We and others have shown that carriers of *TLR5*:c.1174C>T have significantly impaired flagellin sensing [3, 5], and we have reported that *B*. *pseudomallei*-induced IL-10 and IL-6 concentrations in blood from healthy white North American subjects are lower in carriers of the variant while IL-10 levels are reduced in Thai carriers of the variant [5]. To determine whether the effect of this variant on blood cytokine production was flagellin-dependent, we used a markerless in-frame deletion method to create a *B*. *pseudomallei* mutant lacking *fliC*, the gene that encodes flagellin. To assess the ability of the Δ*fliC* mutant to induce TLR5-dependent signaling, we transfected TLR5 into HEK293 cells that only minimally express TLR5 [20]. We stimulated these cells with heat-killed wildtype *B*. *pseudomallei* 1026b or with the heat-killed Δ*fliC* mutant. We confirmed that the Δ*fliC* mutant did not induce any TLR5-dependent NF-κB activation with a luciferase assay (Fig 1). We then stimulated whole blood from healthy subjects with *B*. *pseudomallei* Δ*fliC* and compared IL-10 and IL-6 cytokine levels by *TLR5*:c.1174C>T variant genotype. As was the case following stimulation of blood with wild type *B*. *pseudomallei*, carriers of the variant produced modestly but significantly attenuated IL-10 and IL-6 concentrations upon stimulation with the Δ*fliC* mutant (Fig 2). These results suggested that, besides its established role in modulation of flagellin sensing, the *TLR5*:c.1174C>T variant may alter innate immune responses other than those induced by flagellin.

### Carriers of *TLR5*:c.1174C>T generate reduced cytokine responses to LPS

**Fig 3:**
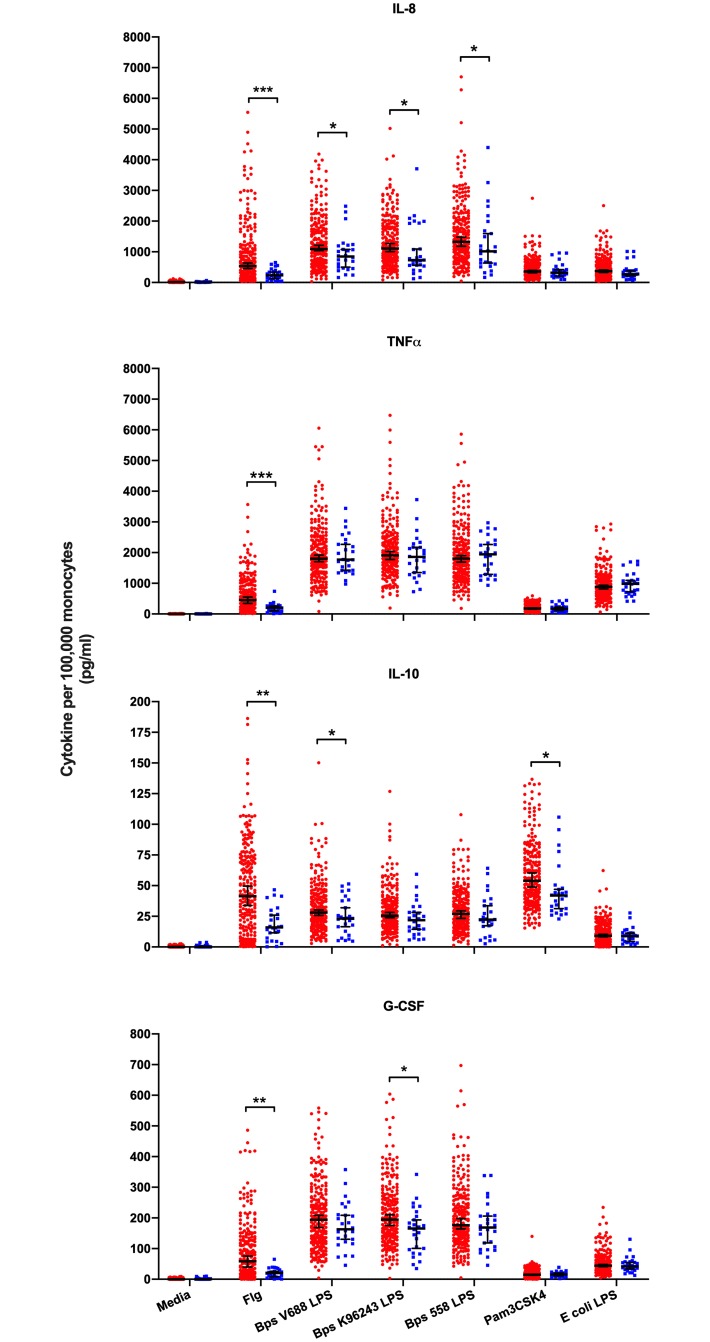
Blood cytokine responses to TLR agonists by *TLR5*:c.1174C>T genotype. Whole blood from healthy Thai subjects was stimulated with media alone, *S*. Typhimurium flagellin 500 ng/ml, *B*. *pseudomallei* V688 LPS 10 ng/ml, *B*. *pseudomallei* K96243 LPS 10 ng/ml, *B*. *pseudomallei* 558 LPS 10 ng/ml, Pam_3_CSK_4_ 100 ng/ml or *E*. *coli* LPS 10ng/ml for 6 hours. IL-8, TNF-α, IL-10 and G-CSF were measured in plasma by multiplex bead assay and normalized to monocyte count. *TLR5*:c.1174C>T genotype (CC, CT, or TT) was determined for each individual by genotyping whole blood DNA. Red dots represent CC (N = 236) and blue dots represent CT or TT (N = 26) genotypes. Superimposed lines represent the median values plus 95% confidence intervals. Statistical analysis was performed by linear regression of log_10_ transformed cytokines assuming a dominant genetic model, adjusting for age, sex and batch. *, P<0.05; **, P≤0.01; ***, P≤0.001. These results are also reported in S1 Table. The media, flagellin, and *E*. *coli* LPS control data have been previously published [5].

We previously demonstrated the importance of *B*. *pseudomallei* LPS in the host response to the bacteria, implicating the LPS-TLR4 axis in melioidosis [7]. To test whether carriage of the *TLR5*:c.1174C>T variant was associated with differential responses to TLR4-dependent stimuli, we measured cytokine and chemokine levels following whole blood stimulation with three different *B*. *pseudomallei* LPS preparations in a large cohort of healthy subjects from Thailand. As control ligands, we also assayed cytokine responses induced by *E*. *coli* LPS, by flagellin and by Pam_3_CSK_4_, a TLR1 agonist. To limit any confounding by other TLR pathway variants, we restricted the analysis to individuals without genetic variation at *TLR4*:c.896A>G and *TIRAP*:c.558C>T. Both of these variants have been associated with susceptibility to infection [21–26]. IL-8, TNF-α, IL-10, and G-CSF concentrations in healthy subjects by *TLR5*:c.1174C>T genotype are shown graphically in Fig 3 and the results of linear regression analysis of the association of genotype and cytokine concentration are shown in S1 Table.

In unstimulated blood from individuals carrying the *TLR5* variant, we observed no difference in released cytokine concentrations. As we have previously reported, in flagellin-stimulated blood from individuals with the variant, all four cytokine responses were uniformly lower [5]. In response to all three *B*. *pseudomallei* LPS, carriers of the variant generated modestly lower IL-8 levels (p<0.05 for all, and all were significant assuming a false discovery rate of 0.10 for all tests performed). In addition, the magnitude of these effects, as assessed by the beta coefficient, was consistently less in LPS-stimulated blood than that in flagellin-stimulated blood. Pam_3_CSK_4_ also induced lower levels of IL-10 in carriers of the variant. Yet the consistent reduction in IL-8 concentrations induced by *B*. *pseudomallei* LPS stimulation of blood of carriers of the *TLR5*:c.1174C>T variant provided supporting evidence that the variant may modulate elements of TLR4-dependent signaling.

### Monocyte expression of *TLR4* mRNA is not changed in carriers of *TLR5*:c.1174C>T

**Fig 4:**
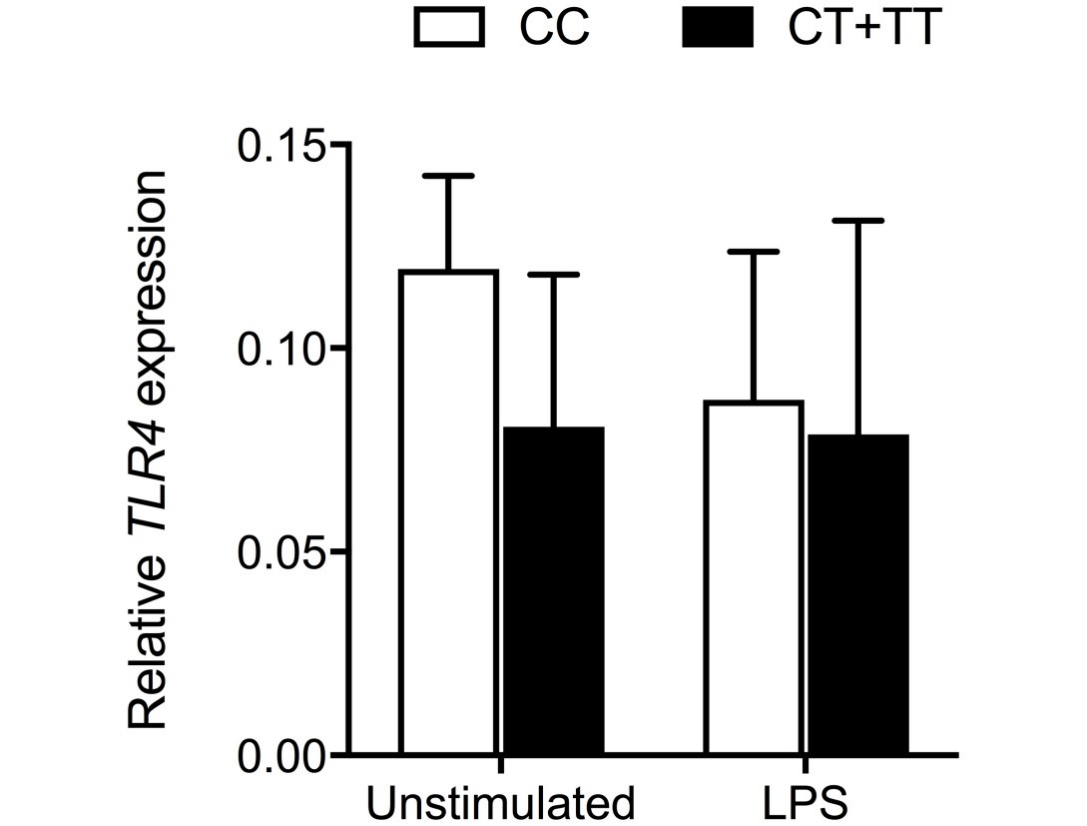
Monocyte expression of *TLR4* is not altered by carriage of *TLR5*:c.1174C>T. Human monocytes isolated from whole blood were left unstimulated or stimulated with *E*. *coli* LPS 10 ng/ml for four hours. RNA was isolated from cell lysates and *TLR4* expression was determined by quantitative RT-PCR and is expressed relative to *GAPDH*. *TLR5*:c.1174C>T genotype (CC, CT, or TT) was determined for each individual by ABI Taqman assay on matched saliva DNA. n = 4 (CC), n = 3 (CT+TT). Mean values ± standard deviation are displayed. P>0.05 for the comparison of relative *TLR4* expression between CC and CT+TT subjects for both conditions using the t test.

We next queried whether the *TLR5*:c.1174C>T-dependent differences in *B*. *pseudomallei* LPS-induced cytokine production observed may be secondary to differences in *TLR4* mRNA expression. We isolated peripheral blood monocytes from individuals with and without the variant and quantified *TLR4* expression. We found that there was no variant-dependent difference in either baseline *TLR4* expression or in *TLR4* expression after stimulation with LPS for four hours (Fig 4). Therefore, it appeared that any effect of the variant on TLR4-dependent signaling is likely to occur down-stream of *TLR4* transcription and mRNA processing.

### TLR5 silencing diminishes LPS-induced TNF-α and IL-8 production in THP1 cells

**Fig 5:**
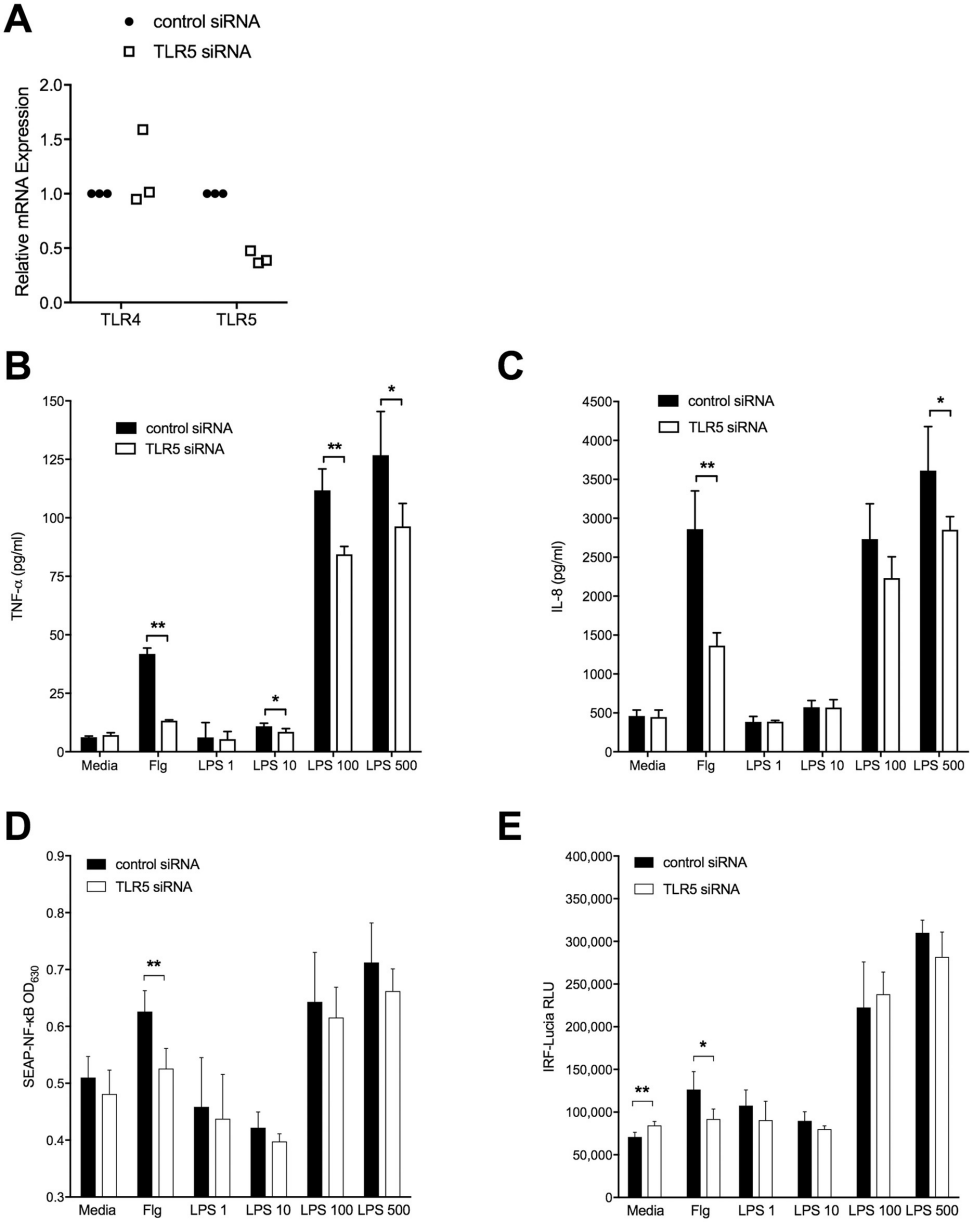
TNF-α and IL-8 production but not NF-κB or IRF3 activity induced by LPS in THP1 cells is impaired upon silencing of TLR5. THP1-Dual Cells were differentiated with Vitamin D3 and plated at 50,000 cells per well were transfected for 48 hours with a negative control siRNA or with siRNA against TLR5 at 5 nM. Cells were unstimulated or stimulated overnight with either *E*. *coli* O111:B4 LPS at varying doses in ng/ml or recombinant *B*. *pseudomallei* flagellin (Flg) at 100 ng/ml as a positive control. A: *TLR4* and *TLR5* mRNA expression were measured by quantitative RT-PCR in cell lysates after 48 hrs of transfection and are expressed relative to ubiquitin C (*UBC)*. Data from three independent experiments are shown, normalized for each experiment. TNF-α (B) and IL-8 (C) concentrations were determined in supernatants the day following stimulation. Transcription factor activation was determined using (D) SEAP-NF-κB and (E) IRF-Lucia luciferase reporter assays, expressed as OD_630nm_ and relative light units, respectively. B-E: Data are displayed as mean ± standard deviations of quadruplicate conditions; one representative example of two or three independent experiments is shown. *, P<0.05; **, P≤0.01.

We then used a gene silencing approach to analyze the effect of TLR5 on LPS-induced cytokine responses. Using siRNA, we knocked down *TLR5* expression in human monocytic THP1-Dual cells. We first confirmed that *TLR5* gene expression was reduced but that *TLR4* gene expression was unchanged (Fig 5A). After stimulating these cells with LPS or with flagellin (as a positive control ligand) we measured cytokines in the cell supernatants. We found that *TLR5* silencing impaired both TNF-α and IL-8 release (Fig 5B & 5C) in response to flagellin as well as in response to LPS. This observation further implicated TLR5 as a regulator of TLR4-driven responses to LPS.

### TLR5 does not definitively alter TLR4-dependent NF-κB or IRF3 activity

**Fig 6:**
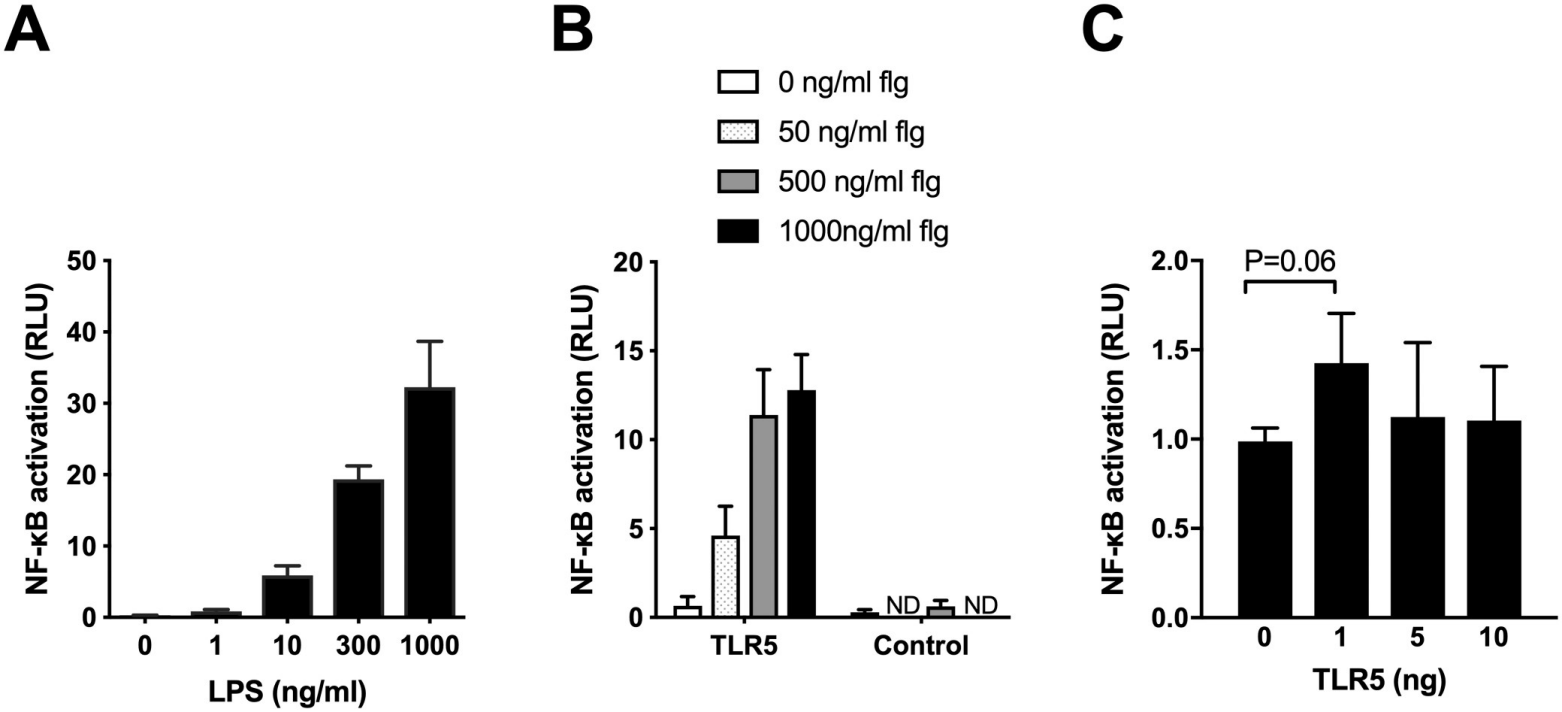
TLR5 does not alter TLR4-dependent NF-κB activity in transfected HEK293 cells. HEK293 cells stably expressing TLR4/MD-2/CD14 were transiently transfected with NF-κB-dependent firefly ELAM luciferase, and *Renilla* luciferase to permit detection of NF-κB activation by luciferase (ratio of ELAM/*Renilla* luciferase signal expressed as relative light units). A: Cells were stimulated with *E*. *coli* LPS for 4 hours. B: Cells were transfected with 5 ng TLR5 and 5 ng of pEF6 (empty vector), and stimulated with *Salmonella* Typhimurium flagellin at various concentrations for 4 hours. For both A and B, mean values ± standard deviations of triplicate conditions are displayed. Representative examples from four independent experiments are shown. C: Cells were transfected with increasing amounts of TLR5 and stimulated with 10 ng/mL *E*. *coli* LPS for 4 hours. Total DNA transfected was kept constant by the addition of pEF6 (empty vector). Data shown are the mean values ± standard deviations of three independent experiments normalized to relative light units for 0 ng TLR5 transfection.

Transcription factors NF-κB and IRF3 mediate LPS-TLR4 signaling. We therefore quantified activation of these transcription factors in the same set of experiments using THP1-Dual cells that express SEAP-NF-κB and IRF-Lucia luciferase reporters. However, we did not detect significant differences in NF-κB or IRF activation after *TLR5* silencing following LPS stimulation in this system (Fig 5D & 5E). In additional experiments, we assessed NF-κB activation after transiently transfecting TLR5 into HEK293 cells stably transfected with TLR4/MD-2/CD14. We first performed a series of pilot experiments to confirm the responsiveness of these cells to LPS and to flagellin based upon an NF-κB luciferase reporter assay (Fig 6A & 6B). We next transfected these cells with increasing quantities of TLR5, stimulated cells with 10 ng/mL LPS, and assessed NF-κB activation. The total amount of DNA transfected in each well was kept constant by adding empty vector as necessary. In this gain-of-function system, we found that addition of 1 ng of TLR5 tended to enhance TLR4-dependent NF-κB activity but at higher doses of TLR5, this effect diminished (Fig 6C). Together, these data do not clearly implicate NF-κB or IRF3 pathways in TLR5 modulation of TLR4-dependent signaling, suggesting that the effect observed on cytokine production may be via other pathways.

## Discussion

In this work we test the hypothesis that TLR5 modulates the flagellin-independent and TLR4-dependent immune response to *B*. *pseudomallei*. We show that selected cytokine responses from individuals carrying the *TLR5*:c.1174C>T nonsense polymorphism are modestly but significantly impaired upon stimulation of blood with *B*. *pseudomallei* lacking flagellin or with *B*. *pseudomallei* LPS. This effect does not appear to be related to altered *TLR4* gene expression in peripheral blood monocytes from carriers of the variant. In monocytic THP1 cells *TLR5* silencing significantly reduces both TNF-α and IL-8 production in response to LPS without an impact on *TLR4* expression or NF-κB or IRF activation. In transfected HEK cells, we did not observe a clear effect of co-transfection of TLR4 and TLR5 on NF-κB activation. Together, these data provide evidence of a modulating effect of TLR5 on the LPS-TLR4 axis but do not identify the underlying mechanism of this effect.

Analysis of human genetic variation offers insights into the relevance of specific proteins in the clinical arena. Our observation of robustly enhanced survival in carriers of the nonsense polymorphism *TLR5*:c.1174C>T infected with *B*. *pseudomallei* [5, 6] prompted us to understand the mechanism underlying this association. As might be expected, *TLR5*:c.1174C>T has a dramatic effect on flagellin-induced cytokine production, as demonstrated in previous work by Hawn *et al*. and by our group [3, 5]. More surprisingly, we now report a flagellin-independent effect of *TLR5*:c.1174C>T on whole blood cytokine production induced by *B*. *pseudomallei* Δ*fliC* or *B*. *pseudomallei* LPS *ex vivo*. While this LPS-dependent effect for the TLR5 variant is not as dramatic as the effect observed upon stimulation with flagellin (based on degree of impairment of cytokine production and range of cytokines impacted), it is nonetheless detectable and significant. Although we have previously reported an association between *TLR5*:c.1174C>T and survival in melioidosis, we have also established the primary importance of LPS in driving innate immune responses to killed *B*. *pseudomallei* [7]. In these studies, the LPS-TLR4 axis was responsible for ~80–90% of the *B*. *pseudomallei*-induced TNF-α release by human monocytes. Our current experiments demonstrating modulation of the LPS-TLR4 axis by *TLR5*:c.1174C>T indicate that the observed association of *TLR5*:c.1174C>T with survival in melioidosis may not be mediated by flagellin-sensing but may instead be mediated by LPS-sensing.

As genetic association studies do not prove causation, we used molecular techniques to address the more general question of whether TLR5 alters TLR4-dependent signaling. We clearly show an effect of TLR5 on LPS-induced/TLR4-dependent TNF-α and IL-8 production after inhibiting *TLR5* expression with siRNA in a monocytic cell line. As we have observed that TLR5 neither detects LPS nor modulates expression of TLR4, CD14, or MD-2 in this system, we speculate that there is an interaction between TLR4 and TLR5 that augments TLR4-dependent signaling. TLR5/TLR4 heteromerization has been demonstrated in transfected COS-1 cells by Mizel et al [8]; consequently, direct heterodimerization of these TLRs may be responsible for the effect we are observing. Alternatively, TLR5 may instead interact with one of the many accessory proteins in the TLR4 pathway to enhance signaling. Further, it is conceivable that the truncation of TLR5 protein in carriers of *TLR5*:c.1174C>T may alter interactions with other innate immune molecules besides TLR4. Whether this truncated protein is membrane-bound or becomes soluble is not clear. To clarify these potential mechanisms, investigation of the protein-protein interactions between human TLR4 and TLR5 (wildtype and variant proteins) is necessary in future studies. Additionally, we could not attribute the observed effects on cytokine production to differential activation of either NF-κB or IRF3, transcription factors that mediate LPS-TLR4 signaling via MyD88-dependent and TRIF-dependent pathways, respectively [27]. Further investigations of other elements of MyD88-dependent pathways such as MAP kinases and transcription factor AP-1 are necessary to better elucidate this mechanism. Additionally, our data indicate that there may be differences in the *TLR5*:c.1174C>T-dependent cytokine response between LPS from *B*. *pseudomallei* and LPS from *E*. *coli*, perhaps implicating distinct and as yet undefined sensing mechanisms and pathways.

While our investigation focused on the interaction of the LPS-TLR4 and flagellin-TLR5 axes in evaluating possible mechanisms attributable to the *TLR5*:c.1174C>T variant in melioidosis, several other mechanisms should be considered. Since we began our studies, other investigators have reported on a role for both human TLR2 and TLR4 in recognition of *B*. *pseudomallei* LPS [28]. This observation raises the possibility of a TLR2-mediated effect on our experiments, an issue that we did not investigate. We also note a modest but significant association between *TLR5*:c.1174C>T genotype and IL-10 induced by TLR2/1 agonist PAM3CSK4 in our whole blood stimulation study. This may simply be due to chance but alternatively may reflect an interaction between TLR2/1 and TLR4. Together, these observations provide corroborating evidence for a complex interplay between TLRs in recognition of bacterial ligands. It is also possible that the phenotypes associated with *TLR5*:c.1174C>T in humans may reflect environmental differences relating to alterations in tonic signaling or microbiota development [29]. These possibilities deserve evaluation in future studies.

Our finding that TLR5 modulates flagellin-independent, TLR4-dependent cytokine release in whole blood and in blood monocytic cells suggests a possible broadened biological significance of the *TLR5*:c.1174C>T polymorphism, with potential implications in melioidosis and in other infections and inflammatory disorders. We acknowledge that the clinical magnitude of the effect may be small. Yet, the *TLR5*:c.1174C>T polymorphism resulting in a non-functional TLR5 protein is fairly common (5% global minor allele frequency). Besides associations with outcomes from melioidosis, outcomes in several other diseases have been associated with this variant. For instance, in carriers of the variant, susceptibility to legionellosis (infection with the Gram-negative flagellated bacterium *Legionella pneumophila*) is increased [3], the risk for Crohn’s disease and systemic lupus erythematosus is decreased, and the body mass index in cystic fibrosis is decreased [30–32]. Such associations underscore the importance of developing a better understanding of the role of TLR5 in innate immune pathways as a potential means of identifying novel therapeutics.

## Supporting information

S1 Table Association of *TLR5*:c.1174C>T in healthy subjects with blood cytokine responses to TLR agonists. (DOCX)
