# Supplementary material for: Flagellin-independent effects of a Toll-like receptor 5 polymorphism in the inflammatory response to Burkholderia pseudomallei
Source: PLoS Negl Trop Dis. 2019 May 8;13(5):e0007354. doi: 10.1371/journal.pntd.0007354 (PMC6527242; doi:10.1371/journal.pntd.0007354)
Supplement: S1 Table — (DOCX) [file pntd.0007354.s001.docx]

Supplemental Data

**Table S1: Association of *TLR5*:c.1174C>T in healthy subjects with blood cytokine responses to TLR agonists**

| Stimulant | Cytokine | Beta coefficient | 95% CI | P value |
| --- | --- | --- | --- | --- |
| None (media) | IL-8 | -0.08 | -0.24 – 0.08 | 0.31 |
|  | TNF-α | 0.06 | -0.20 – 0.33 | 0.64 |
|  | IL-10 | 0.09 | -0.17 – 0.35 | 0.48 |
|  | GCSF | 0.001 | -0.37 – 0.38 | 1.00 |
|  |  |  |  |  |
| Flagellin | IL-8 | -0.43 | -0.63 – -0.23 | **<0.001** |
| 500 ng/ml | TNF-α | -0.41 | -0.61 – -0.21 | **<0.001** |
|  | IL-10 | -0.32 | -0.58 – -0.07 | **0.01** |
|  | GCSF | -0.41 | -0.72 – -0.09 | **0.01** |
|  |  |  |  |  |
| *Bps* V688 LPS | IL-8 | -0.15 | -0.28 – -0.02 | **0.02** |
| 10 ng/ml | TNF-α | 0.01 | -0.08 – 0.09 | 0.89 |
|  | IL-10 | -0.13 | -0.25 – -0.02 | **0.03** |
|  | GCSF | -0.05 | -0.16 – 0.05 | 0.31 |
|  |  |  |  |  |
| *Bps* K96243 LPS | IL-8 | -0.15 | -0.26 – -0.03 | **0.02** |
| 10 ng/ml | TNF-α | -0.04 | -0.12 – 0.04 | 0.39 |
|  | IL-10 | -0.10 | -0.22 – 0.01 | 0.09 |
|  | GCSF | -0.12 | -0.23 – -0.02 | **0.02** |
|  |  |  |  |  |
| *Bps* 558 LPS | IL-8 | -0.14 | -0.26 – -0.01 | **0.03** |
| 10 ng/ml | TNF-α | 0.01 | -0.09 – 0.09 | 0.98 |
|  | IL-10 | -0.10 | -0.22 – 0.02 | 0.11 |
|  | GCSF | -0.06 | -0.16 – 0.04 | 0.23 |
|  |  |  |  |  |
| Pam3CSK4 | IL-8 | -0.07 | -0.19 – 0.05 | 0.25 |
| 100 ng/ml | TNF-α | -0.04 | -0.15 – 0.07 | 0.46 |
|  | IL-10 | -0.10 | -0.18 – -0.01 | **0.03** |
|  | GCSF | 0.04 | -0.13 – 0.20 | 0.68 |
|  |  |  |  |  |
| *E. coli* LPS | IL-8 | -0.11 | -0.24 – 0.02 | 0.10 |
| 10 ng/ml | TNF-α | 0.02 | -0.07 – 0.11 | 0.62 |
|  | IL-10 | -0.02 | -0.25 – 0.19 | 0.85 |
|  | GCSF | -0.004 | -0.12 – 0.11 | 0.95 |
|  |  |  |  |  |

a) Linear regression of log transformed cytokines assuming a dominant genetic model, adjusting for age, sex and batch. N = 236 CC, 26 CT/TT genotypes. P<0.05, uncorrected for multiple comparisons, are shown in bold.

b) Flagellin is from *S.* Typhimurium. *Bps*, *B. pseudomallei*

c) The media, flagellin, and *E. coli* LPS control data have been previously published [5].
